# The Combined Application of the Caco-2 Cell Bioassay Coupled with In Vivo (*Gallus gallus*) Feeding Trial Represents an Effective Approach to Predicting Fe Bioavailability in Humans

**DOI:** 10.3390/nu8110732

**Published:** 2016-11-18

**Authors:** Elad Tako, Haim Bar, Raymond P. Glahn

**Affiliations:** 1 USDA-ARS Robert Holley Center for Agriculture and Health, Ithaca, NY 14853, USA; rpg3@cornell.edu; 2 Department of Statistics, University of Connecticut, Storrs, CT 06269-4120, USA; haim.bar@uconn.edu

**Keywords:** bioavailability, biofortification, iron, beans, pearl millet, screening tools, *Gallus gallus*, Caco-2 bioassay

## Abstract

Research methods that predict Fe bioavailability for humans can be extremely useful in evaluating food fortification strategies, developing Fe-biofortified enhanced staple food crops and assessing the Fe bioavailability of meal plans that include such crops. In this review, research from four recent poultry (*Gallus gallus*) feeding trials coupled with in vitro analyses of Fe-biofortified crops will be compared to the parallel human efficacy studies which used the same varieties and harvests of the Fe-biofortified crops. Similar to the human studies, these trials were aimed to assess the potential effects of regular consumption of these enhanced staple crops on maintenance or improvement of iron status. The results demonstrate a strong agreement between the in vitro/in vivo screening approach and the parallel human studies. These observations therefore indicate that the in vitro/Caco-2 cell and *Gallus gallus* models can be integral tools to develop varieties of staple food crops and predict their effect on iron status in humans. The cost-effectiveness of this approach also means that it can be used to monitor the nutritional stability of the Fe-biofortified crop once a variety has released and integrated into the food system. These screening tools therefore represent a significant advancement to the field for crop development and can be applied to ensure the sustainability of the biofortification approach.

## 1. Introduction

The World Health Organization estimates that approximately one-third of worldwide infant deaths and one half in developing countries can be attributed to malnutrition [1]. More specifically, iron (Fe) deficiency is the most common nutritional deficiency worldwide and a major of infant mortality [1]. Fe deficiency is particularly widespread in low-income countries because of a general lack of consumption of animal products (which can promote non-heme Fe absorption and contain highly bioavailable heme Fe) coupled with a high consumption of a monotonous diet of cereal grains and legumes. Such diets are low in bioavailable Fe due to the presence of phytic acid and certain polyphenols that are inhibitors of Fe bioavailability [2,3]. Recent research also suggests that cellular structures of legumes, such as the cotyledon cell walls, may also be a major factor limiting Fe absorption from legumes [4]. Poor dietary quality is more often characterized by micronutrient deficiencies or reduced mineral bioavailability, than by insufficient energy intake [3,5]. Diets with chronically poor Fe bioavailability which result in high prevalence of Fe deficiency and anemia increase the risk of all-cause child mortalities and also may lead to many pathophysiological consequences including stunted growth, low birth weight, delayed mental development and motor functioning, and others [6,7,8]. Thus, a crucial step in alleviating Fe deficiency anemia is through understanding how specific dietary practices and components contribute to the Fe status in a particular region where Fe deficiency is prevalent.

Biofortification is the breeding of crops to increase their nutritional value, and has primarily focused on increased contents of Fe, Zn and pro-vitamin A [3,9,10]. Biofortification aims to increase the nutrient density in crops during plant growth rather than during processing of the crops into foods [9]. Developing staple food crops for enhanced nutritional quality often requires high throughput methods capable of examining hundreds and sometimes thousands of samples [11,12,13,14]. In general, for Zn and provitamin A, the content of these micronutrients has been more positively correlated with enhanced nutritional quality; whereas for Fe, enhanced content does not always equate to improved nutritional quality [15,16,17,18,19]. Understanding the factors related to the bioavailability of Fe may therefore be the key to developing sustainable Fe-biofortified crops, hence, the development of the appropriate screening tools is vital to properly guide the crop breeding process. For example, research has demonstrated that the Caco-2 cell bioassay [20] is a fast and cost effective approach to screening of hundreds of samples and prior to the selection of the most promising lines to be assessed in vivo [21]. Subsequent feeding trials of the selected lines confirmed the enhanced Fe bioavailability developed via the in vitro screening [22].

The objective of this review is to present recent evidence using a cross-experimental analysis aimed to evaluate the application of the in vitro (Caco-2 cell bioassay) and in vivo (*Gallus gallus*) models as predictive tools for the efficacy of Fe-biofortified crops selected for human efficacy trials. Data from four trials (3 bean and 1 pearl millet) using the above-mentioned screening tools is discussed and analyzed relative to the parallel human efficacy studies [11,15,16,17,23,24,25].

### 1.1. Biofortification of Staple Food Crops as a Sustainable Strategy to Alleviate Dietary Fe Deficiency

As described by Bouis et al., biofortification is the development of micronutrient-dense staple crops using the best traditional breeding practices and modern biotechnology. This multiplier aspect of plant breeding across time and distance makes it cost-effective and sustainable. As the strategy is that nutritionally improved varieties will continue to be grown and consumed year after year, even if government attention and international funding for micronutrient issues fade. Biofortification and commercial fortification, therefore, are highly complementary [3]. In the context of Fe biofortification of staple food crops, recent findings indicate that Fe-biofortification approaches are efficacious in improving iron status in women and children, with the additional potential to benefit Fe-deficient individuals [10]. The first human efficacy trial [26] demonstrated that Fe-biofortified rice improved the iron stores of Fe deficient and nonanemic Filipino women. The high-Fe rice contributed 1.79 mg·Fe/day to the diet in contrast to 0.37 mg·Fe/day from the control rice. The 17% difference in total dietary Fe consumption compared with controls resulted in a modest increase in serum ferritin and total body Fe and no increase in hemoglobin. The greatest improvements in iron status were seen in nonanemic women who had the lowest baseline Fe status and in those who consumed the most iron from rice. Authors concluded that the consumption of biofortified rice, without any other changes in diet, is efficacious in improving iron stores of women with Fe-poor diets in the developing world [26]. Amore recent example, was a study of 136 female Rwandan university students consuming either iron-biofortified or control beans for 4 months, the high-Fe group had significant improvements in hemoglobin (*p* < 0.01), serum ferritin (*p* = 0.015), total body Fe (*p* = 0.024), and VO_2_max (*p* = 0.038) but not energetic efficiency at 60% of maximum work output. There was a significant positive relation between hemoglobin and VO_2_max with analysis of repeated measures taken throughout the study period (*p* < 0.001). These results support previous research that shows that VO_2_max is primarily limited by low hemoglobin and oxygen transport, whereas work efficiency appears to be limited by total body Fe status, which in turn affects oxygen transport and muscular tissue energy metabolism [24]. In another randomized efficacy trial aimed to determine the effects of Fe-biofortified pearl millet on Fe status compared with control pearl millet, results indicated that among children who were iron deficient at baseline, those who received high Fe pearl millet (Fe-PM) were 1.64 times more likely to become Fe replete by 6 months than were those receiving pearl millet of normal Fe density (Control-PM). The effects of Fe-PM on Fe status were greater among children who were Fe deficient at baseline than among children who were not Fe deficient at baseline. Authors concluded that Fe-PM significantly improved Fe status in children by 4 months compared with Control-PM. This study demonstrated that feeding Fe-PM is an efficacious approach to improve Fe status in school-age children and it should be further evaluated for effectiveness in a broader population context [25].

### 1.2. Factors That Limit Fe Bioavailability in a Plant Based Diet Forms of Dietary Fe

There are two types of dietary Fe: nonheme Fe, which is present in both plant foods and animal tissues, and heme Fe, which comes from hemoglobin and myoglobin in animal source foods [27]. Heme Fe is estimated to contribute 10%–15% of total Fe intake in meat-eating populations, but, because of its higher and more uniform absorption (estimated at 15%–35%), it could contribute ≥40% of total absorbed Fe [28,29]. Nonheme Fe is usually much less well absorbed than heme Fe. Historically, scientists have proposed a general hypothesis that nonheme food Fe that enters the common Fe pool in the digestive tract is presumed to be absorbed to the same extent, dependent on the balance between the absorption inhibitors and enhancers and the Fe status of the individual [30,31,32]. Early studies have suggested that not all fortification Fe enters the common pool [3,9,10,13]. However, it is important to note that most of these early studies were conducted using stable or radioisotopes that were added extrinsically to the test meals, with the primary assumption that the extrinsic Fe fully equilibrates with the intrinsic Fe present in the test meal. This assumption has been evaluated and challenged over 30 years ago [33] and has recently been revisited with data suggesting that the primary assumption of extrinsic labelling (i.e., equilibration of extrinsic with intrinsic Fe) is likely to be flawed for staple food crops [34]. Moreover, there is also evidence that cell wall structures in foods such as legumes would also thwart the assumption of equilibration of extrinsic Fe [4]. Thus, it appears that the “common pool” theory of intestinal Fe absorption needs significant revision and certainly additional study with a more modern approach.

#### 1.2.1. Phytate

In plant-based diets, phytate (myo-inositol hexakisphosphate) is a major inhibitor of Fe absorption. The negative effect of phytate on Fe absorption has been shown to be dose dependent and starts at very low concentrations of 2–10 mg/meal [30,35]. The molar ratio of phytate to Fe can be used to estimate the effect on absorption. The ratio should be <1:1 or preferably <0.4:1 to significantly improve Fe absorption in plain cereal or legume-based meals that do not contain any enhancers of Fe absorption, or <6:1 in composite meals with certain vegetables that contain ascorbic acid and meat as enhancers [36,37]. The maximal inhibitory effect of phytate, in the absence of promoters, appears to occur at 10:1 phytate to Fe [38] food processing and preparation methods, which include milling, heat treatment, soaking, germination, and fermentation, can be used to remove or degrade phytate to a varying extent [36,39]. The addition of exogenous phytase or its activation during food processing, or the addition to a meal just before human consumption, has been shown to improve Fe absorption significantly [35,40,41,42].

#### 1.2.2. Polyphenols

Polyphenols occur in various amounts in plant foods and beverages, such as vegetables, fruit, some cereals and legumes, tea, coffee, and wine. The inhibiting effect of polyphenols on Fe absorption has been shown with black tea and herb teas [43,44,45]. At comparable amounts, the polyphenols from black tea were shown to be more inhibiting than the polyphenols from herb teas and wine [45,46]. The fact that polyphenol quantity, as well as type, influences Fe absorption was also shown in a study with spices. Chili, but not turmeric, inhibited Fe absorption in Thai women, although turmeric contained more polyphenols than chili [47]. In cereals and legumes, polyphenols add to the inhibitory effect of phytate, as was shown in a study that compared high and low polyphenol sorghum. After complete phytate degradation, Fe absorption from low-polyphenol sorghum increased significantly, whereas Fe absorption from high-polyphenol sorghum was not improved [48]. In recent years it was suggested that there might be a link between specific polyphenols and the inhibition of Fe bioavailability in Fe biofortified staple food crops (specifically Fe biobortified common beans and pearl millet). For example, the parameters of Fe-status measured in a study aimed to assess Fe bioavailability is Fe biofortified black bean (*Phaseolus vulgaris* L.) in vivo indicated that only a minor increase in absorbable-Fe was achieved with the higher-Fe beans [15,17]. Such results indicate that breeding for higher Fe could concomitantly increase the levels of inhibitory polyphenols and thereby negate the benefit of higher Fe content. In addition, recent research indicates that not all of the polyphenols in bean seed coats are inhibitory, and that some may actually be promoters of [18,19]. For example, in bean seed coats the compounds myricetin, quercetin and quercetin 3-glucoside are definite inhibitors, whereas catechin, kaempferol and kaempferol 3-glucoside are promoters of Fe bioavailability. Regardless, authors have concluded that Fe-biofortified beans remain a promising vehicle for increasing intakes of bioavailable-Fe in human populations that consume high levels of these beans as a dietary staple. These studies emphasize that the bean polyphenol profile must be further evaluated and modified if possible in order to improve the nutritional quality of higher-Fe beans [15]. Moreover, breeding for Fe bioavailability rather than merely for content may be a strategy that has greater impact on Fe biofortification of crops. In another study, aimed to assess the potential of Fe biofortified pearl millet (*Pennisetum glaucum* L.) to deliver more absorbable Fe in vivo, the Fe biofortified peal millet based diet appeared to deliver more absorbable Fe as evidenced by the increased Hb and Hb-Fe status. However, results indicate that the high Fe pearl millet also contained elevated polyphenolic concentrations, which inhibit Fe-bioavailability. As in beans, the authors concluded that these polyphenols-compounds represent potential targets which can perhaps be manipulated during the breeding process to yield improved dietary Fe-bioavailability [16].

### 1.3. In Vitro Assessment of Fe Bioavailability (Caco-2 Cell Bioassay)

In terms of biofortification, target levels for bean Fe concentration have been set at approximately 90 μg/g or higher, which should likely represent a 40 μg/g differential from more typical common bean Fe levels [49,50,51]. That target value is based on calculations that assume the percent bioavailability of the Fe is similar between the normal and enhanced lines, hence delivering more absorbable Fe. It also assumes that normal bean lines are approximately 50 μg/g. Recent research has clearly shown that such Fe levels are common yet often on the low side of what is observed in beans and in other legumes such as lentils [52,53,54]. This assumption is potentially disastrous in terms of plant breeding programs and the planning and ultimately execution of human efficacy trials, as such trials can easily cost $500,000 or more. In addition, the increase in Fe content in the biofortified crop does not always translate to more bioavailable Fe, due to potential Fe bioavailability inhibitors [15,55]. Given this expense and the potential limited nutritional effect on Fe absorption in Fe biofortified staple food crops that may also be rich in phytate and polyphenols, it is prudent to utilize inexpensive screening tools that are validated to predict relative differences in Fe bioavailability prior to conducting costly human efficacy feeding trial and of course prior to the release of such seeds. The in vitro digestion/Caco-2 cell bioassay has been developed specifically to address the need for a cost-effective, high throughput screening tool of Fe bioavailability [20]. This model advanced the field of in vitro Fe bioavailability study as it combined the Caco-2 cell culture (a human intestinal epithelial cell line) with in vitro digestion methods [56], and the high throughput measurement of Caco-2 cell ferritin formation as a measure of Fe uptake. Use of cell ferritin formation negates the need for use of radioisotopes, and the caveats associated with isotopic labelling [34]. It is a sensitive and clear marker of cell Fe uptake as has been shown to it is well known that cells produce ferritin proportionately in response to increases in intracellular iron [52,56,57,58]. Caco-2 cell ferrritin is easily measured via enzyme-linked immunosorbent assay (ELISA) from commercially available kits, the same kits used for human ferritin measurements in clinical practice. Moreover, this method enables measurement of iron availability from foods direct from the producer or supermarket shelf and requires no special preparation for use in the cell model. As evident in the literature, this model appears to be the most widely applied in vitro model in the field of Fe availability. It is a robust model as it has demonstrated the capability to examine a broad range of foods and meal conditions, and produces results that consistently agree with human studies, and also can investigate interactions and factors related to Fe bioavailability in foods [14,15,16,17,18,34,59,60,61,62,63,64,65,66].

### 1.4. In Vivo Assessment of Fe Bioavailability via Poultry (Gallus gallus)

In recent years, we were able to demonstrate that the in vitro Fe bioavailability analysis of selected and biofortified staple food crops was able to predict the potential improvement in Fe status in vivo (*Gallus gallus*) and the delivery of more absorbable Fe for hemoglobin synthesis [11,15,16,17]. The *Gallus gallus* model was shown to exhibit the appropriate responses to Fe deficiency and that it can serve as a model for human Fe bioavailability. More specifically, it is a fast growing animal that is sensitive to dietary mineral deficiencies [60] and is very receptive to dietary manipulations [15,16,17,55,60,61,67,68,69,70,71]. There is also >85% homology between gene sequences of human and chicken intestinal divalent metal transporter 1 (DMT1, the major intestinal Fe transporter), duodenal cytochrome B (DcytB, Fe reductase), ZnT1 (a major intestinal Zn exporter) and Ferroportin (the major intestinal enterocyte Fe exporter), which are key proteins that are essential for Fe and Zn metabolism [72].

In previous years and for in vivo studies using animals, rodents have been the predominant model for Fe bioavailability but appear to have fallen out of favor in recent years due to relatively high efficiency of absorption from foods that have very low availability in humans [73,74]. Piglets have been used as a model but have both strong similarities and differences to human gastrointestinal physiology [74]. The most readily apparent macroscopic difference between human and porcine is intestine length. The small intestine of adult pigs is around 15 to 22 m, whereas the large intestine has an average length of 4 to 6 m [74,75,76,77,78]. In contrast, the small intestine of a human adult averages around 5.5 to 7 m, whereas the large intestine is around 1.5 m [74,77,79,80]. Chickens have a shorter intestinal tract relative to humans (total length is 2.171 m) [81]. The avian digestive system has adaptations designed to facilitate flight. Because birds lack teeth and heavy jaw muscles, food particles are swallowed whole and then reduced in size by the ventriculus and gizzard located within the body cavity [82]. The small intestine is divided into the duodenum, jejunum, and ileum, although these are not distinguishable based on histology or gross observation. There is a distinct duodenal loop, and the yolk stalk is often used as a landmark to separate the jejunum and ileum [81]. The duodenum is the primary Fe absorption site, a feature similar to humans [81].

The modern broiler chicken is a fast-growing animal that is sensitive to dietary deficiencies of trace minerals such Fe [81]. It was also demonstrated that the broiler model mimics the physiological effects of Fe deficiency as reported in other species [60]. As such, it is a relevant model as a source of tissues for in vitro Fe bioavailability studies, in vivo feeding trials, or both [15,16,17,55,60,61,67,68,69,70,71,82]. The poultry model has found a useful niche as an intermediate test of in vivo Fe bioavailability observations in preparation for subsequent human studies [60]. In recent years, the *Gallus gallus* model was used in numerous studies aimed to assess Fe bioavailability, absorption and status in vivo and specifically to assess the effectiveness of Fe biofortified crops (as common bean varieties, pearl millet, sorghum, lentil, maize and wheat) to deliver more absorbable Fe [11,15,16,17] and maintain or improve Fe status. Measurement of food intake and blood hemoglobin coupled with knowledge of food Fe content enables calculation of total body hemoglobin Fe and hemoglobin maintenance efficiency. These parameters allow assessment of Fe status and Fe bioavailability. Other parameters such as gene expression of key proteins of Fe metabolism (i.e., divalent metal transporter 1 (Fe uptake transporter), ferroportin (involved in Fe transport across the enterocyte), and duodenal cytochrome B reductase (reduces Fe at brush border membrane)) and hepatic Fe ferritin content provides a complete picture of the Fe status of the animal both during and at the end of the feeding period. This broad spectrum of physiological and molecular parameters were applied in recent studies and demonstrated an agreement between the in vitro (Caco-2 model) and in vivo (*Gallus gallus* model) observations and the consequences human studies that were aimed to assess the Fe bioavailability and in the same biofortified crops [10,11,15,16,17,25,26].

## 2. Materials and Methods

### 2.1. Ethics Statement

All animal protocols were approved by the Cornell University Institutional Animal Care and Use Committee (protocol name: Intestinal uptake of Fe and Zn in the duodenum of broiler chicken: extent, frequency and nutritional implications; protocol number: 2007-0129).

### 2.2. Animals, Diets and Study Design

For all studies Cornish cross-fertile broiler eggs were obtained from a commercial hatchery (Moyer’s chicks, Quakertown, PA, USA). The eggs were incubated under optimal conditions at the Cornell University Animal Science poultry farm incubator. Upon hatching (hatchability rate = 92%–95%), chicks were allocated into treatment groups (*n* = 14, studies 2 and 4; *n* = 12, studies 1 and 3) on the basis of body weight, gender, and blood hemoglobin concentration (aimed to ensure equal concentration between groups). Study specific dietary treatment groups are detailed in Table 1, Table 2, Table 3 and Table 4. The beans and pearl millet lines that were assessed in these studies were obtained from the International Center for Tropical Agriculture (CIAT, Cali, Colombia) [11,15,17], and the International Crops Research Institute for the Semi-Arid Tropics (ICRISAT, Andrha Pradesh, India) [16]. Samples were shipped to Ithaca, New York in sealed containers imported as grain. Upon arrival, all samples were rinsed in ultra-pure (18 Ω) water and then cooked using an autoclave for 45 min in water and until soft. Samples were then freeze-dried and milled prior to the mixing the diets (for all processing, stainless steel appliances were used). Experimental diets (Table 1) had no supplemental Fe. Chicks were housed in a total confinement building (4 chicks per 1 m^2^ metal cage). The birds were under indoor controlled temperatures and were provided 16 h of light. Each cage was equipped with an automatic nipple drinker and a manual self–feeder. All birds were given ad libitum access to water (Fe content was 0.379 ± 0.012 ppm). Feed intakes were measured daily (as from day 1), and Fe intakes were calculated from feed intakes and Fe concentration in the diets.

Study 1 [11]: Biofortified red mottled beans (*Phaseolus vulgaris* L.) in a maize and bean diet provide more bioavailable iron than standard red mottled beans: Studies in poultry (*Gallus gallus*) and an in vitro digestion/Caco-2 model. Table 1 specifies the bean based diets that were used in the study.

Study 2 [15]: Polyphenolic compounds appear to limit the nutritional benefit of biofortified higher iron black bean (*Phaseolus vulgaris* L.). Table 2 specifies the bean based diets that were used in the study.

Study 3 [16]: Higher iron pearl millet (*Pennisetum glaucum* L.) provides more absorbable iron that is limited by increased polyphenolic content. Table 3 specifies the pearl millet based diets that were used in the study.

Study 4 [17]: Studies of cream seeded Carioca Beans (*Phaseolus vulgaris* L.) from a Rwandan efficacy trial: In vitro and in vivo screening tools reflect human studies and predict beneficial results from iron biofortified beans. Table 4 specifies the bean based diets that were used in the study.

### 2.3. Blood Analysis, Hemoglobin (Hb) Determination, and Tissue Collection

Blood samples were collected weekly from the wing vein (*n* = 14, ~100 µL) using micro-hematocrit heparinized capillary tubes (Fisher, Pittsburgh, PA, USA). Samples were collected in the morning following an 8 h overnight fast. Weekly blood Hb concentrations were determined spectrophotometrically using the cyanmethemoglobin method (H7506-STD, Pointe Scientific Inc., Canton, MI, USA) following the kit manufacturer’s instructions. Fe bioavailability was calculated as hemoglobin maintenance efficiency (HME) [11,15,16,17,55,60]:

(1) $$HME=\frac{\mathrm{Hb}\;\mathrm{Fe},\;\mathrm{mg}\;\left(\mathrm{final}\right)-\;\mathrm{Hb}\;\mathrm{Fe},\;\mathrm{mg}\;\left(\mathrm{initial}\right)}{\mathrm{Total}\;\mathrm{Fe}\;\mathrm{Intake},\;\mathrm{mg}}\times 100$$

where Hb–Fe (index of Fe absorption) = total body hemoglobin Fe. Hb–Fe was calculated from hemoglobin concentrations and estimates of blood volume based on body weight (a blood volume of 85 mL per kg body weight is assumed) [11,15,16,17,55,60]:

(2) Hb–Fe (mg) = B.W.(kg)×0.085 blood/kg ×Hb (g/L) ×3.35 mg Fe/g Hb

At the end of each experiment, birds were euthanized by CO_2_ exposure. The digestive tracts and livers were quickly removed from the carcass and separated into various sections for tissue analysis (small intestine and liver, ~1–2 cm and ~2–3 g, respectively). The samples were immediately frozen in liquid nitrogen, and then stored in a −80 °C freezer until further analysis.

### 2.4. Isolation of Total RNA

Total RNA was extracted from 30 mg of duodenal (proximal duodenum) tissue using the Qiagen RNeasy Mini Kit (Qiagen Inc., Valencia, CA, USA) according to the manufacturer’s protocol. All steps were carried out under RNase free conditions. RNA was quantified by absorbency at 260–280 nm. Integrity of the 28S and the 18S rRNA was verified by 1.5% agarose gel electrophoresis followed by ethidium bromide staining [11,15,16,17,55,60,69,70,71,82,83,84].

### 2.5. DMT–1, DcytB and, Ferroportin Gene Expression Analysis

As previously described [11,15,16,17,55,60,69,70,71,82,83], PCR was carried out with primers chosen from the fragments of chicken duodenal tissues (DMT-1 gene (GeneBank database; GI 206597489) (forward: 5’-AGCCGTTCACCACTTATTTCG-3’; reverse: 5’-GGTCCAAATAGGCGATGCTC-3’), DcytB gene (GI 20380692) (forward: 5’-GGCCGTGTTTGAGAACCACAATGTT-3’; reverse: 5’-CGTTTGCAATCACGTTTCCAAAGAT-3’) and Ferroportin gene (GI 61098365) (forward: 5’-GATGCATTCTGAACAACCAAGGA’; reverse: 5’-GGAGACTGGGTGGACAAGAACTC-3’). Ribosomal 18S was used to normalize the results (GI 7262899) (forward: 5’-CGATGCTCTTAACTGAGT-3’; reverse: 5’-CAGCTTTGCAACCATACTC-3’)). All PCR products were separated by electrophoresis on 2% agarose gel stained with ethidium bromide, and quantified using the Quantity One 1-D analysis software (Bio-Rad, Hercules, CA, USA).

### 2.6. In Vitro Fe Bioavailability Assessment

An in vitro digestion/Caco-2 cell culture model was used to assess in vitro Fe bioavailability [4,11,15,16,17,18,20,21,34,52,55,59,60,61,62,66,69,70,71,82,83,84]. With this method, the cooked and freeze dried crop samples, additional meal plan components and the formulated diets were subjected to simulated gastric and intestinal digestion. 0.5 g of the freeze dried cooked beans and diet samples were utilized for each replication (*n* = 6) of the in vitro digestion process.

### 2.7. Harvesting of Caco-2 Cells for Ferritin Analysis

The protocols used in the ferritin and the total protein contents analyses of Caco-2 cells were similar to those previously described [4,11,15,16,17,18,20,21,34,52,55,59,60,61,62,66,69,70,71,82,83,84]. Caco-2 cells synthesize ferritin in response to increases in intracellular Fe concentration. Therefore, we used the ratio of ferritin/total protein (expressed as ng ferritin/mg protein) as an index of the cellular Fe uptake. All glassware used in the sample preparation and analyses was acid washed.

### 2.8. Ferritin and Fe in the Liver, Electrophoresis, Staining and Measurement of Gels

Liver ferritin and liver Fe quantification were conducted as previously described [11,15,16,17,68,82,85,86]. The gels were scanned with a Bio-Rad densitometer, and measurements of the bands were conducted using the Quantity-One 1-D analysis program (Bio-Rad, Hercules, CA, USA). All assays were conducted in duplicates for each animal in both biofortified and standard treatment groups (*n* = 14).

### 2.9. Polyphenol Extraction

Isolated beans seed coats were prepared by wrapping whole beans in de-ionized water-soaked paper towels until seed coats began to wrinkle and separate from cotyledons. Seed coats were then removed with forceps, dried, and ground to a coarse powder with mortar and pestle. To one gram of ground material, 8 mL of methanol:water (50:50 v:v) was added. The slurry was vortexed for one minute, placed on an orbital shaker for 25 min, then placed in a 30 °C sonication water bath for 15 min, vortexed again for one minute, and centrifuged at 4000× *g* for 12 min. The supernatant was filtered with a 0.2 µm Teflon syringe filter and stored for later use in a −20 °C freezer [15,17,18].

### 2.10. Ultra Performance Liquid Chromatography–Mass Spectrometry (UPLC–MS) Analysis of Polyphenols

Seed coat extracts and polyphenol standards were analyzed with an Agilent 1220 Infinity UPLC coupled to an Advion expressionL compact mass spectrometer (CMS). 2 μL samples were injected and passed through an Acquity UPLC BEH Shield RP18 1.7 µm 2.1 × 100 mm column (Waters) at 0.35 mL/min. The column was temperature-controlled at 45 °C. The mobile phase consisted of water with 0.1% formic acid (solvent A) and acetonitrile with 0.1% formic acid (solvent B). Polyphenols were eluted using linear gradients of 86.7% to 77.0% A in 0.5 min, 77.0% to 46.0% A in 5.5 min, 46.0% to 0% A in 0.5 min, hold at 0% A for 3.5 min, 0% to 86.7% A in 0.5 min, and hold at 86.7% A for 3.5 min for a total 14 min run time. From the column, flow was directed into a variable wavelength UV detector set at 278 nm. Flow was then directed into the source of an Advion expression LCMS (Advion Inc., Ithaca, NY, USA) and ESI mass spectrometry was performed in negative ionization mode using selected ion monitoring with a scan time of 50 msec for each of 8 polyphenol masses of interest. Capillary temperature and voltages were 300 °C and 100 V, respectively. ESI source voltage and gas temperature were 2.6 kV and 240 °C respectively. Desolvation gas flow was 240 L/h. LC and CMS instrumentation and data acquisition were controlled by Advion Mass Express software. Identities of polyphenols in bean samples were confirmed by comparison of *m*/*z* and LC retention times with authentic standards. Polyphenol quantification was achieved by the use of standard curves and integration of UV absorption peak areas [15,17,18].

### 2.11. Determination of Phytic Acid Concentration in the Diet Samples

Dietary phytic acid (phytate)/total phosphorus was measured as phosphorus released by phytase and alkaline phosphatase, following the kit manufacturer’s instructions (*n* = 5, K-PHYT 12/12, Megazyme International, Wicklow, Ireland) [11,15,16,17].

### 2.12. Statistical Analysis

**Figure 1:**
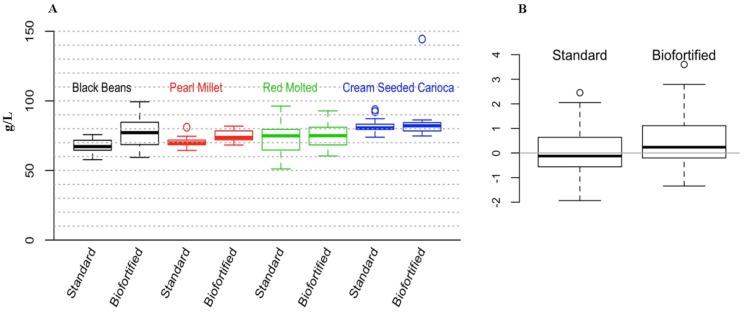
Blood hemoglobin concentrations (g/L). (**A**) Experimental treatment differences (mean ± SEM, *p* < 0.05); (**B**) overall standardized and cross experimental standard vs. biofortified treatment groups.

**Figure 2:**
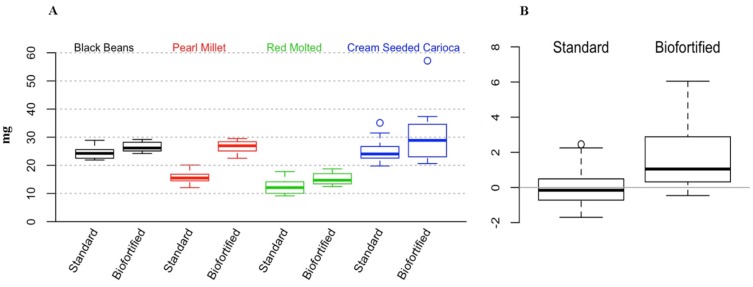
Total body Hb-Fe (mg). (**A**) Experimental treatment differences (mean ± SEM, *p* < 0.05); (**B**) overall standardized and cross experimental standard vs. biofortified treatment groups.

**Figure 3:**
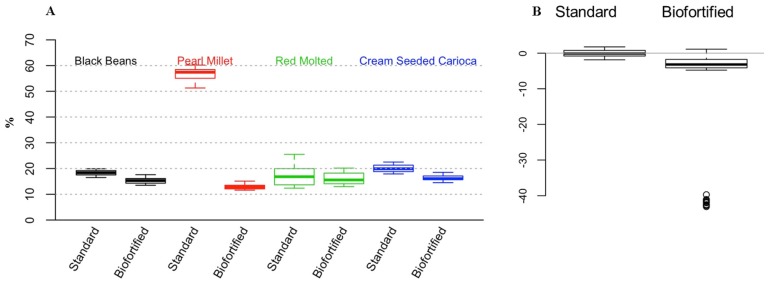
Hemoglobin maintenance efficiency (%). (**A**) Experimental treatment differences (mean ± SEM, *p* < 0.05); (**B**) overall standardized and cross experimental standard vs. biofortified treatment groups (*p* < 0.05).

**Figure 4:**
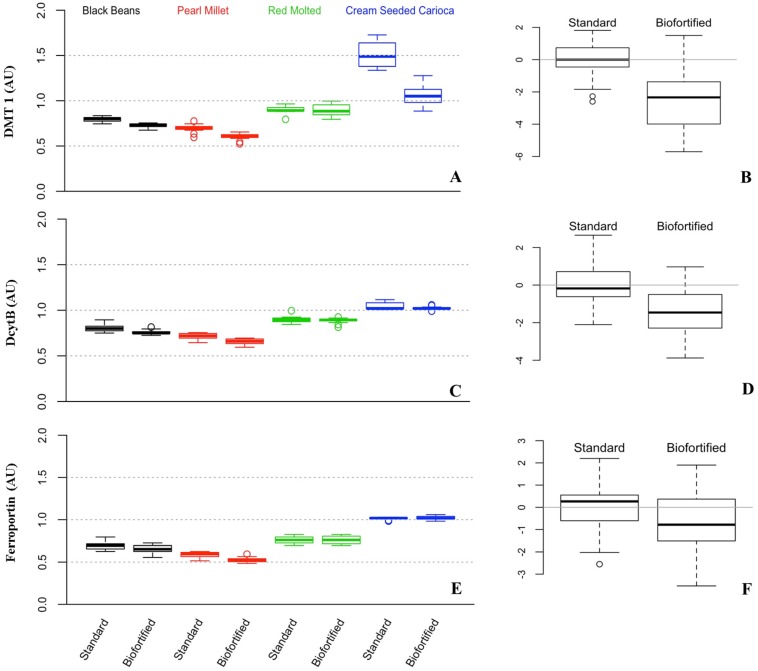
Duodenal mRNA expression of DMT1, DcytB and Ferroportin. Changes in mRNA are shown in relative expression of 18S rRNA in arbitrary units (mean ± SEM, AU, *p* < 0.05). (**A**,**C**,**E**) Experimental treatment differences; (**B**,**D**,**F**) overall standardized and cross experimental standard vs. biofortified treatment groups.

For the cross experimental analyses that are presented in this review, we used a factorial analysis of variance (ANOVA) to test whether the type of diet (2 levels: standard, biofortified) or experiment (4 levels: Black Beans, Pearl Millet, Red Mottled Beans, Cream Seeded Carioca Beans), or their interaction was a statistically significant factor. The response variables were: Blood Hb, Total Body Hb-Fe, Hemoglobin Maintenance Efficiency (HME), gene expression of Fe related transporters and enzyme (Divalent Metal Transporter 1 (DMT1), Duodenal Cytochrome B (DcytB), and Ferroportin). We controlled the probability of Type I error at the 0.05 level and used Tukey’s HSD method to account for multiple testing. To validate the ANOVA assumptions, we tested for normality using the Shapiro-Wilk test, and tested for homogeneity of variances using Levene’s test. In order to test whether the overall response variables were significantly different between the standard and the biofortified diets, we standardized the data as follows. In each of the four experiments we considered the mean response in the standard diet group as the reference for that experiment and subtracted it from each observation. Thus, in Figure 1B, Figure 2B, Figure 3B and Figure 4B,D,F, the overall mean for the standard diet is 0. For each experiment/diet combination we standardized the mean-centered responses by dividing all the observations by the corresponding standard deviation. Then, to test whether the over mean response is different between the two treatments (standard vs. Fe biofortified), we combined the standardized results from the four experiments and performed a t-test. We controlled the probability of Type I error at the 5% level, and used the Bonferroni correction to account for multiple testing. For HME, Total body Hb-Fe, DMT, and DCytB, there is a significant difference between the two diets across all four experiments. Results for the in vitro and liver Fe and ferritin contents were analyzed by ANOVA using the general linear model procedures of SAS software (SAS Institute Inc., Cary, NC, USA). Differences between treatments were compared by Tukey’s test and values were considered statistically different at *p* < 0.05 (values in the text are means ± SEM).

## 3. Results

In this section, cross experimental data are presented of in vitro and in vivo parameters that were previously published and aimed to assess Fe bioavailability in Fe biofortified Red Mottled Beans (*Phaseolus vulgaris* L.) [11], Black Bean (*Phaseolus vulgaris* L.) [15], Cream Seeded Carioca Beans (*Phaseolus vulgaris* L.) [17] and Pearl Millet (*Pennisetum glaucum* L.) [16].

### 3.1. Cross Experimental In Vitro Assessments of Fe Bioavailability

The in vitro digestion/Caco-2 cell culture model was used to evaluate Fe bioavailability from the tested diets by measuring ferritin in the cells (i.e., a measure of cell Fe uptake) following exposure to digests of the samples [4,11,15,16,17,18,20,21,34,52,55,59,60,61,62,66,69,70,71,82,84]. The results showed that in the red mottled beans study [11], ferritin concentrations were significantly higher (*p* < 0.05) in cells exposed to the biofortified bean based diet vs. the standard bean based diet, as well as being higher in cells exposed to the Fe biofortified beans vs. the standard beans only (*n* = 6, Table 5). These results indicate greater amounts of bioavailable Fe in the Fe biofortified beans and diet. In the black beans study [15], ferritin concentrations were not significantly different in cells exposed to the biofortified bean based diet vs. the standard bean base diet (*n* = 6, Table 5). The lack of difference in Fe bioavailability was likely due to the increased polyphenolic content in the Fe biofortified beans that limits their nutritional benefit. In the pearl millet study [16], ferritin concentrations were significantly higher (*p* < 0.05) in cells exposed to the biofortified pearl millet based diet vs. the standard pearl millet based diet, as well as higher in cells exposed to the Fe biofortified pearl millet vs. the standard pearl millet only. These results indicate greater amounts of bioavailable Fe in the Fe biofortified pearl millet and diet. (*n* = 6, Table 5). In the cream seeded carioca beans study [17], ferritin concentrations were significantly higher (*p* < 0.05) in cells exposed to the biofortified bean based diet vs. the standard bean based diet, as well as higher in cells exposed to the Fe biofortifies beans vs. the standard beans only. These results indicate greater amounts of bioavailable Fe in the Fe biofortified beans and diet. (*n* = 6, Table 5). Overall, the in vitro model provided useful and vital information in regards to the Fe bioavailability in the tested sample. It demonstrated the ability to identify lines with greater bioavailable Fe that was confirmed in the in vivo trials.

### 3.2. Cross Experimental In Vivo Assessment of Fe Bioavailability, Absorption and Status

Figure 1, Figure 2, Figure 3 and Figure 4 depict the distributions of Blood hemoglobin concentrations (g/L), Total body Hb-Fe (mg), Hemoglobin maintenance efficiency (%), and Duodenal mRNA expression of DMT1, DcytB and Ferroportin. In each plot, panel A shows the distribution of the corresponding measurement for each combination of experiment (BB, PM, RM, and CSC) and diet (Standard and Biofortified). The distributions are represented as “boxplots”. The upper and lower sides of the box correspond to the 75th and 25th percentiles, respectively. The horizontal line in each box represents the median of the observations, and the “whiskers” on both sides depict the interquartile range. Points outside the whiskers are possible outliers. Panels A show the distributions in the original units (g/L, mg, %, and AU). To generate Panel B in each plot, we standardized the data so that in each experiment/diet combination the mean is set to 0 and the standard deviation is set to 1. The purpose of the standardization is to put the measurements in all experiments on the same scale. In particular, if one experiment resulted in much higher/lower values than in the other experiments (as in the case of Hemoglobin maintenance efficiency in the standard diet), then combining it with other experiments without standardizing first, would skew the aggregate distribution. The combined standardized measurements for each diet type are then used to produce the two boxplots in panel B of each plot.

#### 3.2.1. Blood Hb

Results indicated that in the black bean study [15], hemoglobin concentrations were only significantly higher in the Fe biofortified group vs. the standard group at week 6 (*p* < 0.05). In the pearl millet [16], hemoglobin concentrations were higher at week 5 and beyond (*p* < 0.05) in the Fe biofortified group vs. the standard group. In the red mottled bean study [11], hemoglobin concentrations were not significantly higher in the Fe biofortified group vs. the standard group. In the cream seeded carioca beans study [17], hemoglobin concentrations were not significantly different at any time point when compared to the standard group.

#### 3.2.2. Total Body Hb-Fe

Results indicated that in the in the black bean study [15], the increase in total body Hb Fe from the beginning of the study to the end of the 6th week was significantly greater in the biofortified group vs. the standard group (25.5 ± 0.8 mg and 23.3 ± 0.6 mg, respectively, *p* < 0.05, Figure 2A). In the pearl millet study [16], the increase in total body Hb Fe from the beginning of the study to the end of the 6th week was significantly greater in the biofortified group vs. the standard group (25.6 ± 1.4 mg and 14.4 ± 0.8 mg, respectively, *p* < 0.05, Figure 2A). In the red mottled bean study [11], the increase in total body Hb Fe from the beginning of the study to the end of the 4th week was significantly greater in the biofortified group vs. the standard group (12.6 ± 0.7 mg and 10.2 ± 0.4 mg, respectively, *p* < 0.05, Figure 2A). In the cream seeded carioca bean study [17], the increase in total body Hb Fe from the beginning of the study to the end of the 6th week was significantly greater in the biofortified group vs. the standard group (26.6 ± 1.3 mg and 21.4 ± 1.1 mg, respectively, *p* < 0.05, Figure 2A).

Total body Hb–Fe is a physiological biomarker that is commonly used as an index of Fe status [11,15,16,17,55,60]. Higher Hb-Fe values suggest improved Fe status. In the studies discussed here, this parameter appears to be the most useful indicator of the amount of Fe delivered from a biofortified crop during the feeding period. The results presented here indicated that in all studies, the total body Hb Fe values were higher in the Fe biofortified treatment groups vs. the standard treatment groups (*p* < 0.05, Figure 2B).

#### 3.2.3. Hemoglobin Maintenance Efficiency (HME)

Results indicated that in the black beans study [15], significant increases in HME were measured on days 14, 21 and 28 of the experiment in the group receiving the standard bean based diet vs. the biofortified bean based diet (*p* < 0.05, Figure 3A). In the pearl millet study [16], significant increases in HME were measured at all-time points in the group receiving the standard bean based diet vs. the biofortified bean based diet (*p* < 0.05, Figure 3A). In the red mottled bean study [11], significant increases in HME were measured on days 14 and 21 of the experiment in the group receiving the standard bean based diet vs. the biofortified bean based diet (*p* < 0.05, Figure 3A). In the cream seeded carioca bean study [17], significant increases in HME were measured at all-time points in the group receiving the standard bean based diet vs. the biofortified bean based diet (*p* < 0.05, Figure 3A).

Hemoglobin maintenance efficiency represents the calculated amount (%) of dietary Fe that is incorporated into hemoglobin, and is therefore a relatively conservative measure of fractional dietary Fe bioavailability [11,15,16,17,55,60,63,64,65,67]. The HME calculation must therefore be considered in the conjunction with dietary Fe concentration, Fe absorption requirements and Fe status of the animal consuming the diet. For example, in the pearl millet study of Figure 3, the dietary Fe levels of the standard pearl millet group were well below the amount of dietary Fe required by the animal model; hence, the HME values were much higher, likely representing an adaption of the animal to increase Fe absorption to meet metabolic needs. Conversely, the biofortified pearl millet likely contained much more Fe than necessary for the animal to meet metabolic needs, hence HME would be much lower.

### 3.3. Gene Expression of Fe Related Transporters and Enzyme (Divalent Metal Transporter 1 (DMT1), Duodenal Cytochrome B (DcytB), and Ferroportin)

Results indicated that in the black bean study [15], gene expression analysis of duodenal DMT1, DcytB, and Ferroportin, with results reported relative to 18S rRNA, indicated no significant differences in mRNA expression of DMT1, DcytB and Ferroportin in the standard group compared to the Fe biofortified group (*n* = 6, *p* > 0.05, Figure 4A,C,E). In the pearl millet study [16], gene expression analysis of duodenal DMT1, DcytB, and Ferroportin, with results reported relative to 18S rRNA, revealed increased mRNA expression of DMT1, DcytB and Ferroportin in the standard group compared to the Fe biofortified group (*n* = 6, *p* < 0.05, Figure 4A,C,E). In the red mottled bean study [11], gene expression analysis of duodenal DMT1, DcytB, and Ferroportin, with results reported relative to 18S rRNA, revealed increased mRNA expression of DMT1, DcytB and Ferroportin in the standard group compared to the Fe biofortified group (*n* = 6, *p* < 0.05, Figure 4A,C,E). In the cream seeded carioca beans study [17], gene expression analysis of duodenal DMT1, DcytB, and Ferroportin, with results reported relative to 18S rRNA, revealed increased mRNA expression of DMT1 in the standard group relative to the Fe biofortified group (*p* < 0.05). However, no significant differences in DcytB and Ferroportin expression were observed between treatment groups (*n* = 6, *p* > 0.05, Figure 4A,C,E).

The gene expression of key Fe related transporters (DMT1 and Ferroportin), and enzyme (DcytB) is used as a molecular indicator of the cellular response to Fe status [11,15,16,17,55,63,64,67,68,69,70,71,82,87]. Increased duodenal expression of the Fe transporters, divalent-metal-transporter-1, and ferroportin occurs in humans with iron deficiency, as these genes are upregulated to increase the BBM efficiency to absorb a more limited available pool of luminal Fe [88,89,90]. Results indicated that there was a gene expression up regulation of DMT1, DCYTB and Ferroportin in the standard treatment groups vs. Fe biofortified (studies 1 and 3). In addition, DMT1 expression was up regulated in the standard group vs. the Fe biofortified (study 4). Overall, DMT1, DcytB and Ferroportin expression is reactive to dietary Fe status, as its relative expression is up regulated under the more Fe deficient conditions (Figure 4B,D,F; *p* < 0.05).

### 3.4. Ferritin and Fe in the Liver

Liver Fe and liver ferritin concentrations are used as additional physiological indicators for Fe status in vivo [11,15,16,17,55,67,68,85,86]. The avian ferritin corresponded to a weight of approximately 470 to 500 kDa [85,86]. No significant differences in liver Fe or liver ferritin were measured between the treatment groups (Red mottled beans; Black beans and Pearl millet studies, *n* = 6, *p* > 0.05) [11,15,16]. In the cream seeded carioca beans study [17], although no significant differences were measured in liver ferritin were measured, increased liver Fe concentrations were detected in the Fe biofortified group vs. the standard group (Table 6).

### 3.5. Total Concentration of Polyphenols in the Diets

Concentrations of the polyphenols known to be most prevalent in black beans [15] were measure and are shown in Table 7. In vitro data now indicates that kaempferol, epicatechin, Kaempferol 3-glucoside are potential Fe uptake promoters. However, myricetin and quercetin 3-glucoside are known to be potent inhibitors that can overwhelm the promoter effects even at lower concentrations [18,19]. These inhibitors were significantly higher in the Fe biofortified black beans, and thus may have limited the nutritional benefit of the higher Fe content. The concentration of the five most prevalent polyphenols found in the seed coat of cream seeded carioca beans [17] is shown in Table 7. Both Kaempferol 3-glucoside, and quercetin 3-glucoside were significantly elevated in the Fe biofortified beans (*p* < 0.05). Kaempferol 3,4-dihydroxybenzoin acid, and catechin were not significantly different between bean varieties (*p* > 0.05). Finally, phenolic analysis of the pearl millet samples [16] detected three specific mass-to-charge ratios (*m*/*z*), one of which significantly higher in the Fe biofortified pearl millet variety (AU, *p* < 0.05). The elevated mass (*m*/*z* = 431.09) corresponds to 15 possible candidate glycosylated phenolic compounds. The glucones of these compounds, as well as their purported effect on Fe absorption and bioavailability can be found in Table 8.

## 4. Discussion

### 4.1. The Correlation between Screening Tools (In Vitro and In Vivo Models) for Fe Bioavailability and Human Efficacy Studies

The primary goal of this review is to provide definitive cross experimental analyses to demonstrate the link between specific in vitro (Caco-2 bioassay; in vitro digestion/Caco-2 cell bioassay) and in vivo (*Gallus gallus*) models designed to assess Fe bioavailability from foods [11,15,16,17,20,55]. The in vitro digestion/Caco-2 cell bioassay was originally developed with the primary intention of fulfilling a need for a screening tool to rank Fe bioavailability among varieties of staple food crops [52,98]. However, it has been shown to be even more robust and capable of assessing Fe bioavailability from any food or meal, and evaluate factors that can influence Fe bioaccessibility from food [56,99,100,101,102,103]. The *Gallus gallus* model [11,15,16,17,55,60,67,68,69,70,71] was developed as it became clear that an intermediate level of in vivo study could be highly useful and cost-effective to confirm in vitro results, enable a long term feeding trial (versus a single or multiple meals) and provide added confidence and refinement of experimental objectives for varieties to be advanced to human efficacy studies. For staple food crops, it is important to recognize that plant breeders usually work in small amounts of seed for variety development, often less than 1 kg. The poultry model usually requires 25–35 kg for a 6–7 week feeding trial, and human studies often require several hundred kilograms. Generating the amount of seed necessary to conduct a poultry feeding trial obviously represents significant time, cost and effort, which is further amplified for the human efficacy study. Confidence should be high in the effectiveness of the foods when the study progresses to human feeding and these models are designed to fulfil that requirement.

It is also important to note the relative costs and throughput of the above models compared to human studies. For the in vitro model, considering only technician salary and supplies (as institution costs vary), a skilled technician dedicating only 50% of their time can easily process 2000 samples per year at an approximate cost of $26 per sample. That same technician could also conduct 2–3 studies per year with the poultry model, at 6–7 weeks per study, and easily evaluate 10–12 samples at salary and supply cost of about $85,000. It is not unreasonable for a human efficacy study, comparing only 2 samples to cost most than $300,000 [34,104]. It is clearly a cost-effective and natural extension of these models to apply them in series to evaluation of varieties, diets and meal plans of human efficacy studies.

The cross experimental statistical analyses of the studies described in this review make a strong case for utilizing the Caco-2 cell bioassay and the poultry model as tools to develop and evaluate crop varieties for improved Fe nutrition. Historically, Fe biofortification has focused solely on increasing Fe content of the crops, setting target values for beans at 90–100 μg/g, which are about 30–50 μg/g higher than what is assumed to be in most of the prominent lines of beans [54]. This target value for beans assumes that Fe bioavailability remains the same and was calculated to provide a nutritionally significant increase in the amount of bioavailable Fe. The models of this study demonstrate that for the 2 out of the 3 bean studies, this amount of Fe did indeed provide nutritional benefit, which is in agreement with the corresponding human efficacy studies [23,24,25,26,105]. In addition, these screening tools also have the capacity to provide a fast and cost-effective monitoring of Fe biofortified crops once they are released to farmers and dispersed into the food system. Such monitoring will likely be needed to ensure the biofortification effect over time, as the specific crop dietary Fe bioavailability may change. Overall, we conclude that Fe biofortified bean varieties and pearl millet remain a promising vehicle for increasing intakes of bioavailable Fe in African and Andean populations that consume these beans.

### 4.2. Strategy: Tailoring a Specific Diet

In studies of Fe biofortification, there is a clear need and advantage to evaluate biofortified lines of staple food crops, both individually and in the context of the diet for which they are consumed [11,15,16,17,63,64,65]. The screening tools presented in this paper are capable of doing so. Moreover, they can also be applied to identify processing and or cooking steps that can affect Fe content and bioavailability and that may negate or enhance the effectiveness of the biofortified crop [16,17,59,106]. The studies discussed here, demonstrate how the in vitro digestion/Caco-2 cell model and the *Gallus gallus* model of Fe bioavailability could be applied to the design of a human efficacy study, conducted in advance, and thus assess the Fe bioavailability of Fe biofortified versus standard staple food crop. For example, the carioca cream seeded bean based diets that were used were specifically formulated according to the menus that were offered in the Rwandan human efficacy study [23]. The subjects of this study were fed a cafeteria style meal plan where bean consumption was strictly monitored with periodic monitoring of choices and consumption of the other food products. The main dietary components in addition to beans were potato, pasta, tomato sauce, banana, basmati rice, cabbage and arrange sweet potato [23] (Table 1).

### 4.3. Use of a Caco-2 Cell Bioassay in Identification and Characterization of Specific Polyphenolic Compounds That May Inhibit the Nutritional Benefit of the Fe Biofortified Staple Food Crop

Polyphenolic compounds are considered to be inhibitors of Fe bioavailability [11,56,107]. Because they are presumed to act in a similar manner, total polyphenols have commonly measured via the Folin-Ciocalteu colorimetric assay. In a recent study, the content of polyphenolic compounds in white and black beans was measured and the effect of individual polyphenols on Caco-2 cell iron uptake was characterized [18,19]. Analysis of seed coat extracts by LC-MS revealed the presence of a range of polyphenols in black bean, but no detectable polyphenols in white bean. Extracts from black bean seed coats strongly inhibited iron uptake. Examination of the eight most abundant black bean seed coat, non-anthocyanin polyphenols via Caco-2 cell assays showed that four (catechin, 3,4-dihydroxybenzoic acid, kaempferol, and kaempferol 3-glucoside) clearly promoted iron uptake and four (myricetin, myricetin 3-glucoside, quercetin, and quercetin 3-glucoside) inhibited iron uptake. The four inhibitors were present in 3-fold higher total concentration than the promoters (143 ± 7.2 vs. 43.6 ± 4.4 μM), consistent with the net inhibitory effect observed for black bean seed coats. The ability of some polyphenols to promote Fe uptake and the identification of specific polyphenols that inhibit Fe uptake suggest a potential for breeding bean lines with improved Fe nutritional qualities [19].

It would appear that identification of polyphenols that can promote Fe bioavailability raises the possibility of producing breeding lines with enhanced nutritional quality. The results reported by [18] were obtained using an in vitro cell culture system, and it remains to test these in vitro effects in an in vivo approach. It is interesting that a human study of the effects of several polyphenols determined that, whereas gallic acid and tannic acid inhibited Fe absorption, catechin produced no such inhibition [18,43]. This observation suggests that, even if polyphenol promotion of iron uptake in cell culture does not translate to promotion in vivo, breeding for reduced levels of inhibitory polyphenols as well as enhanced levels of promoting polyphenols may yield more nutritious staple foods.

In the cream seeded carioca bean study [17], significant differences in phytic acid concentration were observed between the biofortified and standard bean varieties (Table 1). Further, the beans seed coat polyphenols analysis detected two polyphenols that were significantly higher in concentration in the Fe biofortified carioca bean, quercetin 3-glucoside and kaempferol 3-glucoside. In fact, measurable levels of quercetin 3-glucoside were not observed in the standard bean variety (Table 4). Previously, quercetin 3-glucoside and kaempferol 3-glucoside have been found in measureable quantities in beans [15,16,17,18,19,108,109], and were shown to complex ferric Fe (Fe+3), thus limiting the bioavailability of dietary Fe [91,110]. Acute and chronic quercetin ingestion has also been shown to inhibit duodenal Fe utilization [110]. A similar response has been noted in Caco-2 cells exposed to quercetin [111,112]. Further, increased concentration of the flavonol kaempferol 3-glucoside has been previously detected in Fe biofortified black beans [11], and has also been shown to inhibit in vitro Fe bioavailability in red and pinto beans [57], however, recent evidence indicates that in lower concentrations, it functions as a promotor of Fe bioavailability (in vitro) (Table 2) [19]. The purported mechanism for the Fe inhibitory effects of kaempferol and quercetin can be attributed to their chemical structures as they are able to chelate metallic ions, thus forming insoluble complexes with Fe3+ and limiting its uptake by the enterocyte [91,93,95,111,112]. However, recent evidence indicated that Kaempferol elevates Fe bioavailability in vitro and in lower concentrations [18,19]. As has been previously suggested, breeding towards an increased Fe content in beans may also increase the polyphenol, phytic acid, and similar “antinutrient” (e.g., tannins) content which in turn may limit the nutritional benefit of the Fe biofortified crops [2,3,16,17]. However, since many polyphenols act as strong cellular antioxidant and anti-carcinogenic compounds [113,114,115], an important goal of future research should be to identify and manipulate concentrations of specific families, even perhaps individual compounds, which display Fe inhibitory properties. Doing so, these health-promoting polyphenols may be largely retained while the effects of Fe inhibition could be limited. Overall, continued research by using the dual in vitro and in vivo screening guiding tools is needed to confirm our findings, and assess the feasibility of such a plant breeding strategy [105,115].

LC/MS analysis of the pearl millet lines indicated on a *m*/*z* ratio of 431.09 corresponding to 15 unique parent polyphenolic aglycones (Table 2), significantly elevated in the Fe biofortified pearl millet compared to the standard Fe pearl millet. The plant metabolites identified belong to chemical families including flavones, flavonols, isoflavones, and anthocyanins, many of which have been shown to inhibit Fe absorption [91,92,93,94,95,96], (Table 3) either by direct mineral chelation and Fe efflux or, in the case of the phytoestrogen isoflavones, by modulating membrane Fe receptor expression and thus affecting Fe homeostasis [96]. For example, [94] elucidated antioxidant effects of baicalein through Fe-binding in a physiologically-relevant in vitro model. It was determined that baicalein bound Fe^2+^ more strongly than ferrozine, a well-known Fe^2+^ chelator. These results are consistent with others [116,117,118] who have found a variety of phenolic and polyphenolic compounds, namely kaempferol, luteolin, and apigenin, in different varieties of millet (mainly E. coracana, an utricles millet). For a detailed review of relevant phenolic compounds found in millet, please see [119,120].

In agreement with numerous others, our data further supports the notion that phytic acid and polyphenolic compounds, such as quercetin, quercetin glucosides, myricitin and myricitin glucoside, may likely be responsible for limiting the effects of significantly increasing Fe concentration in the biofortified beans (Table 2), and other potential compounds in Fe biofortified pearl millet (Table 3). Since these biofortified crops increased bioavailable Fe in vivo, we believe that it remains a central priority to further evaluate and if possible to modify the polyphenol profile of beans and pearl millet in order for their ingestion to confer an optimal Fe status.

## 5. Conclusions

**Figure 5:**
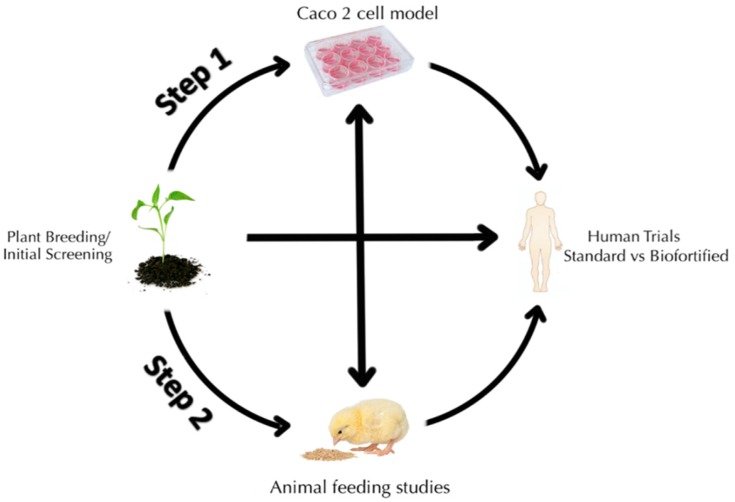
Schematic diagram depicting Fe bioavailability screening of biofortified staple food crops. Step 1, assessing Fe bioavailability in vitro (the Caco-2 cell bioassay), this model allows rapid and cost effective screening of hundreds of samples. Step 2, selection of most promising lines and tailoring the appropriate and specific diet that is relevant to the target population to be assessed in a long term in vivo feeding trial (*Gallus gallus*). This two-step screening process is employed in advance of human efficacy studies to refine experimental design, evaluate the biofortified food in the context of the targeted food system. This approach not only predicts but can cost-effectively monitor the Fe-biofortified crop once released to farmers.

The current review highlights agreement between in vitro*,* animal studies [11,15,16,17] and their parallel human efficacy trials. Measurable nutritional effects were observed as a result of consuming Fe biofortified cream seeded carioca beans [10,23,24,106] and pearl millet [10,25]. This summary clearly supports the in vitro and in vivo screening tools as an effective two step system to guide crop development for Fe biofortification, and evaluate the crop varieties within the food systems for which they are targeted. In addition, these screening tools also have the capacity to cost-effectively monitor Fe biofortified crops once they are released to farmers and dispersed into the food system. Such monitoring will likely be needed to ensure the biofortification effect (Figure 5).

## Figures and Tables

**Table 1:** Composition of experimental diets.

Ingredient Low-Fe Bean Diet High-Fe Bean Diet		
g/kg (by Formulation)		
High-Fe Beans (71 µg Fe/g), NUA35	−	600
Low-Fe Beans (49 µg Fe/g), CAL96	600	−
Corn	200	200
Corn oil	30	30
Dry skim milk	100	100
Vitamin/mineral premix (no Fe) ^1^	70	70
dl-Methionine	2.5	2.5
Choline Chloride	0.75	0.75
Total (g)	1000	1000
Selected components	mean ± SEM, *n* = 5 (by analysis)	
Fe (µg Fe/g) ^2^	42.9 ± 1.2 a	54.6 ± 0.9 b
Total Phenols (gallic acid, µg/g) ^3^	103.5 ± 5.5 a	101.8 ± 6.1 a
Phytate:Fe molar ratio ^3^	8.28 ± 9.2 a	8.59 ± 1.06 a

^1^ Vitamin and mineral premix provided/kg diet (330,002 Chick vitamin mixture; 230,000 Salt mix for chick diet; *Dyets* Inc., Bethlehem, PA, USA); ^2^ Iron concentrations in the diets were determined by an inductively-coupled argon-plasma/atomic emission spectrophotometer (ICAP 61E Thermal Jarrell Ash Trace Analyzer, Jarrell Ash Co., Franklin, MA, USA) following wet ashing; ^3^ Method for determining phenol concentrations and phytate contents are described in the materials and methods section; a,b: Within a row, means without a common letter are significantly different, *p* < 0.05.

**Table 2:** Composition of experimental diets ^1–3^.

Ingredient Standard Bean Diet Biofortified Bean Diet		
g/kg (by Formulation)		
Biofortified Beans (88 µg Fe/g),MIB465	−	400
Standard Beans (59 µg Fe/g), DOR500	400	−
Corn	350	350
Corn oil	30	30
Dry skim milk	100	100
Corn starch	46.75	46.75
Vitamin/mineral premix (no Fe) ^1^	70	70
dl-Methionine	2.5	2.5
Choline Chloride	0.75	0.75
Total (g)	1000	1000
Selected components	mean ± SEM, *n* = 5 (by analysis)	
Fe (µg Fe/g) ^2^	39.4 ± 0.2 b	52.9 ± 0.9 a
Phytate:Fe molar ratio ^3^	8.25 ± 0. 65 a	8.95 ± 0.72 a

^1^ Vitamin and mineral premix provided/kg diet (330,002 Chick vitamin mixture; 230,000 Salt mix for chick diet; *Dyets* Inc., Bethlehem, PA, USA); ^2^ Iron concentrations in the diets were determined by an inductively-coupled argon-plasma/atomic emission spectrophotometer (ICAP 61E Thermal Jarrell Ash Trace Analyzer, Jarrell Ash Co., Franklin, MA, USA) following wet ashing; ^3^ Method for determining phenol concentrations and phytate contents are described in the materials and methods section; a,b: Within a row, means without a common letter are significantly different, *p* < 0.05.

**Table 3:** Composition of the experimental diets ^1–3^.

Ingredient	*High-Fe* (Biofortified)	*Low-Fe* (Standard)
g/kg (by Formulation)		
*High-Fe* Pearl millet (84.9 µg/g Fe), ICTP 8203	750	−
*Low-Fe* Pearl Millet (25.9 µg/g Fe), DG 9444	−	750
Skim milk, dry	100	100
dl-Methionine	2.5	2.5
Corn starch	47.5	47.5
Corn oil	30	30
Choline chloride	0.75	0.75
Vitamin/mineral premix (no Fe)	70	70
Total (g)	1000	1000
Selected components	mean ± SEM, *n* = 5 (by analysis)	
Dietary Fe concentration (µg/g)	78.6 ± 0.51 a	22.1 ± 0.52 b
Phytic Acid (µg/g)	9940 ± 1380 a	10,500 ± 230 a
Phytate:Fe molar ratio ^3^	10.7 ± 0.55 b	40.2 ± 0.35 a

^1^ Vitamin and mineral premix provided/kg diet (330,002 Chick vitamin mixture; 230,000 Salt mix for chick diet; *Dyets* Inc., Bethlehem, PA, USA); ^2^ Iron concentrations in the diets were determined by an inductively-coupled argon-plasma/atomic emission spectrophotometer (ICAP 61E Thermal Jarrell Ash Trace Analyzer, Jarrell Ash Co., Franklin, MA, USA) following wet ashing; ^3^ Method for determining phenol concentrations and phytate contents are described in the materials and methods section; a,b: Within a row, means without a common letter are significantly different, *p* < 0.05.

**Table 4:** Composition of the experimental bean based diets ^1–5^.

Ingredient	Fe Content^1^	Standard Bean Diet	Biofortified Bean Diet
µg Fe/g, (*n* = 5, by Analysis) g/kg (by Formulation)			
Biofortified-Fe Beans, SMC	106.1 ± 0.204	–	346
Standard-Fe Beans, G4825	57.10 ± 0.145	346	–
Basmati Rice	0.290 ± 0.006	135	135
Pasta (non-enriched)	11.48 ± 0.358	70	70
Potato flakes	10.26 ± 0.061	215	215
Banana Chips	7.510 ± 0.521	115	115
Cabbage	16.32 ± 0.400	30	30
Tomato powder	39.92 ± 1.187	16	16
Orange sweet potatoes	26.90 ± 0.611	73	73
Vitamin/mineral premix (no Fe) ^2^	0.00 ± 0.00	70	70
dl-Methionine	0.00 ± 0.00	2.5	2.5
Vegetable oil	0.00 ± 0.00	30	30
Choline chloride	0.00 ± 0.00	0.75	0.75
Total (g)		1000	1000
Selected components		mean ± SEM, *n* = 5 (by analysis) ^4^	
Dietary Fe concentration (µg/g)		33.7 ± 0.80 b	48.7 ± 1.50 a
Phytic acid (µg/g) ^3^		10,605 ± 742 b	13,793 ± 1172 a
Phytate:Fe molar ratio		15.43 ± 0.85 a	10.95 ± 0.65 b

^1^ Iron concentrations in the diets were determined by an inductively-coupled argon-plasma/atomic emission spectrophotometer (ICAP 61E Thermal Jarrell Ash Trace Analyzer, Jarrell Ash Co., Franklin, MA, USA) following wet ashing; ^2^ Vitamin and mineral premix provided/kg diet (330,002 Chick vitamin mixture; 230,000 Salt mix for chick diet; *Dyets* Inc., Bethlehem, PA, USA); ^3^ Method for determining phenol concentrations and phytate contents are described in the materials and methods section; ^4^ The specific Rwandese dietary formulation that was used in the study (Table 1) was achieved by a close consultation and approval of the HarvestPlus nutritionist team, and was based on the menus that were used during the human efficacy trial [23,24]. a,b: Within a row, means without a common letter are significantly different, *p* < 0.05.

**Table 5:** Ferritin concentration in Caco-2 cells exposed to samples on the tested staple crop only (whole seed), and the crop based diets.

	Tested Sample ^1^	Cream Seeded Carioca Beans Study [17]	Black Beans Study [15]	Red Mottled Beans Study [11]	Pearl Millet Study [16]
Food Crop					
Ferritin (ng/mg of Protein)					
Standard variety only		2.86 ± 0.14 b	2.31 ± 0.11 c	7.82 ± 0.75 d	1.22 ± 0.05 c
Fe Biofortified variety only		4.40 ± 0.14 a	2.19 ± 0.14 c	30.6 ± 2.08 a	2.61 ± 0.36 a
Standard variety based diet		1.96 ± 0.05 d	2.97 ± 0.10 b	11.2 ± 0.97 c	1.47 ± 0.27 bc
Fe Biofortified variety based diet		2.73 ± 0.23 bc	2.75 ± 0.09 b	15.7 ± 1.05 b	2.46 ± 0.13 a
Cell baseline ^2^		2.53 ± 0.07 c	3.28 ± 0.13 a	4.06 ± 0.37 e	1.54 ± 0.12 b

^1^ Caco-2 bioassay procedures and preparations of the digested samples were previously described [11,15,16,17]; ^2^ Cells were exposed to only MEM (minimal essential media) without added food digests and Fe; a,b: Within a column, means without a common letter are different, *p* < 0.05.

**Table 6:** Ferritin protein and the iron concentration in the liver ^1^.

	Treatment Group	Ferritin (µg/g Wet Weight)	Iron (µg/g Wet Weight)
Red Mottled Bean study [11]	Standard	409 ± 12 a	39.5 ± 3.5 a
	Fe biofortified	425 ± 18 a	48.1 ± 4.2 a
Black bean study [15]	Standard	282 ± 12 a	27.2 ± 1.7 a
	Fe biofortified	293 ± 11 a	33.1 ± 2.2 a
Pearl Millet study [16]	Standard	277 ± 7.1 a	19.3 ± 2.7 a
	Fe biofortified	285 ± 8.5 a	25.2 ± 3.9 a
Cream Seeded Carioca bean study [17]	Standard	284 ± 13 a	45.5 ± 3.4 b
	Fe biofortified	315 ± 22 a	62.6 ± 5.7 a

^1^ Atomic mass for iron used for calculations defined as 55.8 g/mol; a,b: within a column (and for each study), means with a common letter are not significantly different (*p* < 0.05, means ± SEM).

**Table 7:** Concentrations of prevalent polyphenols observed in cream seeded carioca beans and black beans seed coat ^1^ (µmol/g) [15,17].

Bean Variety	Compound	“Biofortified Fe”	“Standard Fe”	Putative In Vitro Effect on Fe Absorption/Bioavailability [15,17,18,19]
Black beans	Caffeic acid	0.060 ± 0.0009 b	0.026 ± 0.004 a	↑
	Gallic acid	0.125 ± 0.0088 a	0.103 ± 0.018 a	↑
	Ferulic acid	0.153 ± 0.011 a	0.163 ± 0.020 a	↓
	Kaempferol	0.005.0 ± 0.0001 a	0.00 ± 0.00 b	↑
	Catechin	0.669 ± 0.0311 a	0.367 ± 0.025 b	↑
	Myricetin	0.024 ± 0.0017 a	0.012 ± 0.004 b	↓
	Kaempferol 3-glucoside	0.198 ± 0.0107 a	0.019 ± 0.005 b	↑
	Quercetin 3-glucoside	0.239 ± 0.0203 a	0.046 ± 0.007 b	↓
Cream seeded carioca beans	3,4-dihydroxybenzoic acid	0.211 ± 0.02 a	0.198 ± 0.002 a	↑
	Catechin	0.179 ± 0.004 a	0.175 ± 0.02 a	↑
	Quercetin 3-glucoside	0.085 ± 0.01 a	0.00 ± 0.00 b	↓
	Kaempferol 3-glucoside	0.302 ± 0.007 a	0.206 ± 0.008 b	↑
	Kaempferol	0.015 ± 0.001 a	0.015 ± 0.001 a	↑

^1^ Analysis procedures of beans samples are described in the materials and methods sections; a,b: Within a row, means without a common letter are different (*n* = 6, *p* < 0.05). ↓ Decrease of Fe bioavailability/absorption in vitro; ↑ Increase of Fe bioavailability/absorption in vitro.

**Table 8:** Aglycone of polyphenolic compounds corresponding to an *m*/*z* = 431.09 highly-enriched in the High-Fe pearl millet [16].

Class	Compound	Putative In Vitro Effect on Fe Absorption/Bioavailability	Citation
**Flavones**	Apigenin	↓	[91,92,93]
	Baicalein	↓	[94,95]
	Luteolin	↓	[92]
	Norwogonin	*	
	Scutellarein	*	
	5,7,2’-Trihydroxyflavone	*	
	7,3’,4’-Trihydroxyflavone	*	
	7,3’,4’,5’-Tetrahydroxyflavone	*	
**Flavonol**	Galangin	↓	[96]
	Kaempferol	↓ ↑	[18,19,92]
**Isoflavones**	Dihydrodaidzein	↓	[92]
	Genistein	↓	[93,97]
	Trihydroxyisoflavone	*	
	6,7,4’-trihydroxyisoflavone	*	
**Anthocyanins**	Pelargonidin	↓	[98]

* As of the writing of this paper, no data on the putative effects of these compounds relating to Fe absorption/bioavailability exist; ↓ Decrease of Fe bioavailability/absorption in vitro; ↑ Increase of Fe bioavailability/absorption in vitro.

## Abbreviations

Fe	iron
Hb	hemoglobin
HME	hemoglobin maintenance efficiency
DMT1	divalent metal transporter 1
DcytB	duodenal cytochrome B
UPLC–MS	ultra performance liquid chromatography–mass spectrometry
